# Locally Advanced Adenoid Cystic Carcinoma of the Right Submandibular Gland With Tuberculosis: A Case Report and Review of the Literature

**DOI:** 10.7759/cureus.68658

**Published:** 2024-09-04

**Authors:** Komal V Dadgal, Swapnil Mohod, Vikrant V Jadhav, Tushar Sontakke, Neha Rahul

**Affiliations:** 1 Oral Medicine and Radiology, Sharad Pawar Dental College and Hospital, Datta Meghe Institute of Higher Education and Research, Wardha, IND; 2 Dentistry, Dr. Panjabrao Alias Bhausaheb Deshmukh Memorial Medical College, Amravati, IND; 3 Orthodontics and Dentofacial Orthopedics, Sharad Pawar Dental College and Hospital, Datta Meghe Institute of Higher Education and Research, Wardha, IND; 4 Medicine, Jawaharlal Nehru Medical College, Datta Meghe Institute of Higher Education and Research, Wardha, IND; 5 Radiation Oncology, Siddharth Gupta Memorial Cancer Hospital, Datta Meghe Institute of Higher Education and Research, Wardha, IND; 6 Radiation Oncology, Jawaharlal Nehru Medical College, Datta Meghe Institute of Higher Education and Research, Wardha, IND

**Keywords:** cylindroma, histopathological diagnosis, radiographic investigations, adenoid cystic carcinoma, submandibular gland, salivary gland tumor

## Abstract

Adenoid cystic carcinoma (ACC) is an aggressive form of salivary gland cancer that mostly affects the accessory, parotid, and submandibular salivary glands. This tumor is characterized by slower development, perineural invasion, and possible local recurrence in clinical and pathological findings. A male patient, aged 71, who was from a remote area, appeared with a lesion affecting the right submandibular gland and had been experiencing discomfort in the same region for four months. Following a biopsy and the histological confirmation of ACC in the right submandibular gland, the tumor was widely excised locally.

## Introduction

A salivary gland tumor known as adenoid cystic carcinoma (ACC) can develop in salivary glands, both major and minor. It is predominantly seen among women [1]. While it is rare in the parotid, ACC primarily originates from the submandibular gland and the minor salivary glands. The palate, tongue, lacrimal glands, paranasal sinuses, external auditory canal, nasopharynx, and larynx are the sites of origin in the head and neck region [2-5]. Secretory glands in other tissues, including the Bartholin's glands, prostate, uterine cervix, tracheobronchial tree, lungs, breast, epidermis, and esophagus, can also give rise to ACC. According to historical research by Ranger et al., the hard and soft palates are the oral cavity's most involved areas. Approximately 400 glands are dispersed throughout the uvula, hard and soft palates, and both [4].

It appears as a firm consistency nodule that is fixed upon palpation and covered with intact mucosa during the clinical evaluation. The ACC is a tumor that expresses myoepithelial and ductal epithelial cells, two types of malignancies that resemble the salivary glands' intermediate ducts. The parenchyma of the tumor may have a solid, cribriform, or tubular morphologic appearance [6-8]. From a pathological perspective, salivary gland malignancies are among the most complicated categories of tumors. There are 24 known malignant histotypes based on the WHO's 2005 classifications, nearly all of which have distinct morphological, genetic, and clinical characteristics [5]. The WHO classified 23 malignant histotypes in their 2017 classification. There is no other location in the head and neck where the diversity of malignant tumors is as great. A competent pathologist is required for the diagnosis due to the variety and rarity of some of them [2]. Even though ACC is one of the most prevalent carcinomas of the salivary gland, it is not common in many pathology departments, which makes it challenging for general pathologists to become knowledgeable in the area [9-11].

## Case presentation

**Figure 1:**
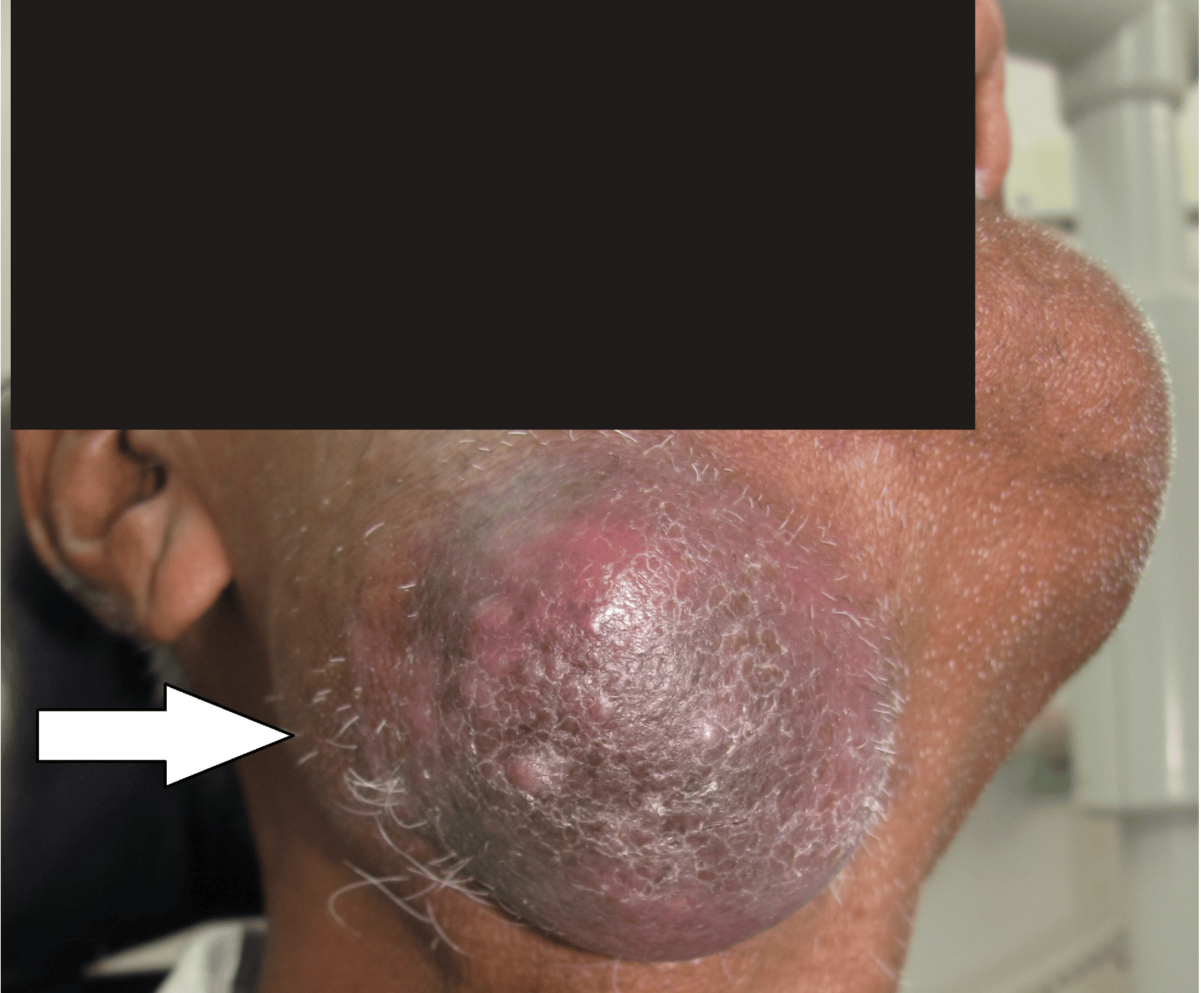
The extraoral swelling involving the right submandibular region

A 71-year-old male patient reported to the outpatient department of oral medicine and radiology with the chief complaint of swelling in the right posterior region of the lower jaw for four months. The patient was apparently alright four months ago when he started noticing swelling, which was initially small but gradually progressed to the current size. The patient gave a history of associated pain that aggravates on mastication. The patient reported that he developed similar swelling in the cervical region one year ago, for which he took ayurvedic medications and got relief. The patient gave a history of self-exfoliation of multiple teeth. He also has a history of habitual cigarette smoking with a frequency of three to four cigarettes per day for 15 years. There was no history of similar presentations in any of the family members. The overall general examination of the patient revealed no abnormality. On extraoral examination, the extension of the swelling was superoinferior from the corner of the mouth to 4 cm below the right lower border of the mandible. The anteroposterior extension of the lesion was from the level of the right lip commissure on the face to the angle of the mandible. The size of the swelling was approximately 6 cm x 5 cm with a brownish-blue color. The surface of the swelling was shiny and smooth. On palpation, all the inspectory findings were confirmed. The consistency of the swelling was firm to hard with raised local temperature. The swelling was tender to palpate, fixed to the underlying bone, and non-fluctuant (Figure 1).

**Figure 2:**
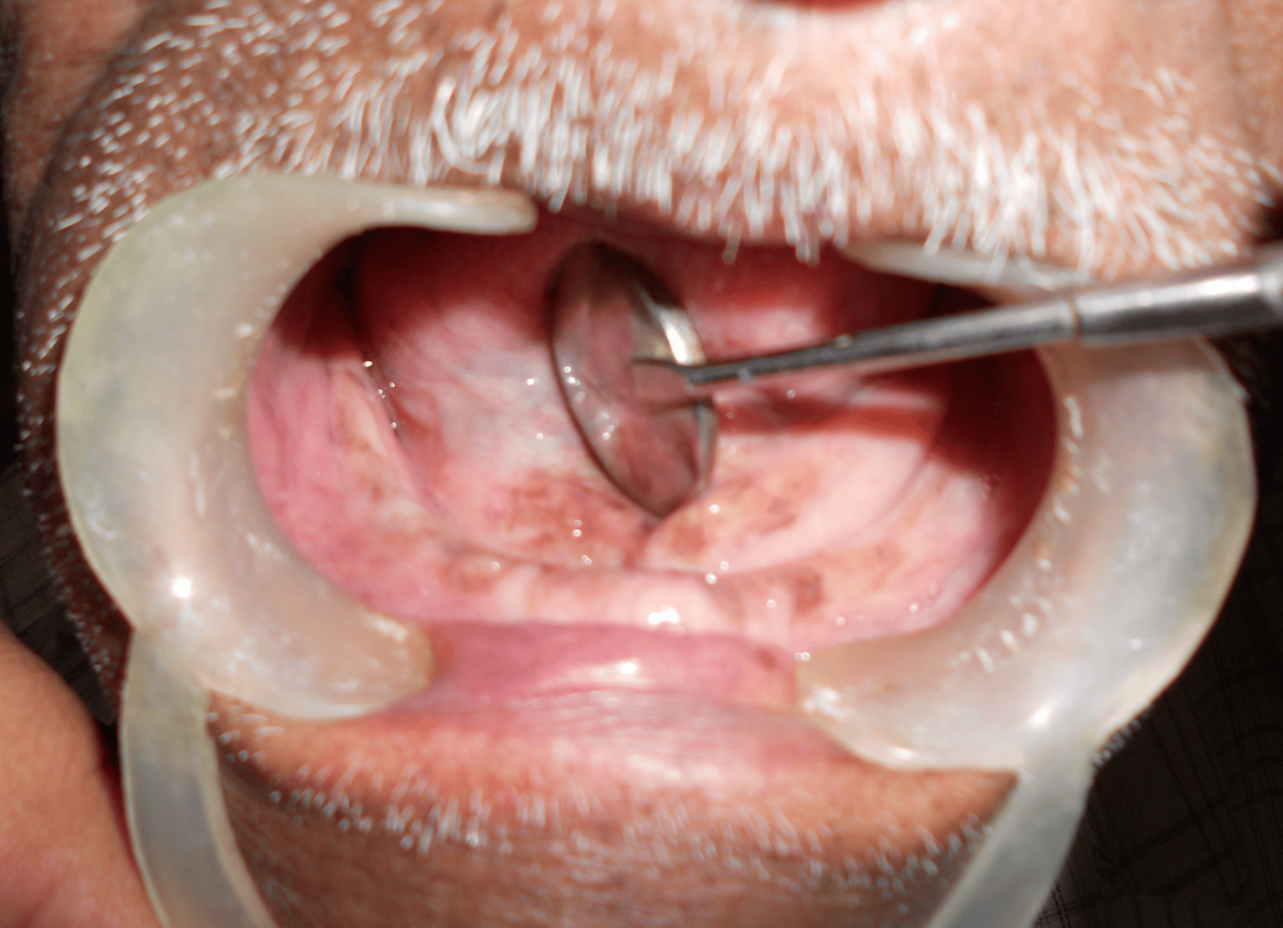
The complete edentulous lower arch with no evidence of intraoral swelling in the lower right posterior region of the jaw

Submandibular lymph nodes were palpable. Intraorally, on inspection, there was no evidence of swelling. The upper and lower arches were edentulous. On palpation, there was a single diffuse swelling present in the lingual sulcus on the right side in regions 44 to 46, extending anteroposteriorly from regions 44 to 47 and superoinferiorly from the crest of the alveolar ridge; the lower aspect was not palpable. The consistency of the swelling was firm to hard and tender to palpate. A single bony spicule was palpable in region 47 (Figure 2).

**Figure 3:**
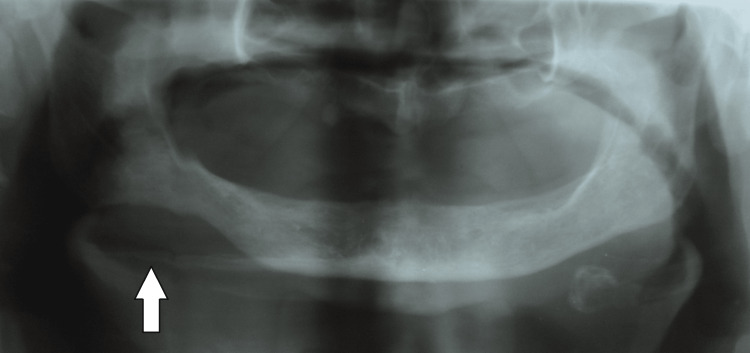
Evidence of a well-defined radiolucency in the right mandibular region

The patient was advised for radiographic investigations and fine needle aspiration cytology (FNAC) of the swelling. The orthopantomogram (OPG) of the patient revealed edentulous upper and lower arches. There was evidence of a well-defined radiolucency in the right mandibular region extending from the right parasymphyseal region to the right angle of the mandible (Figure 3).

**Figure 4:**
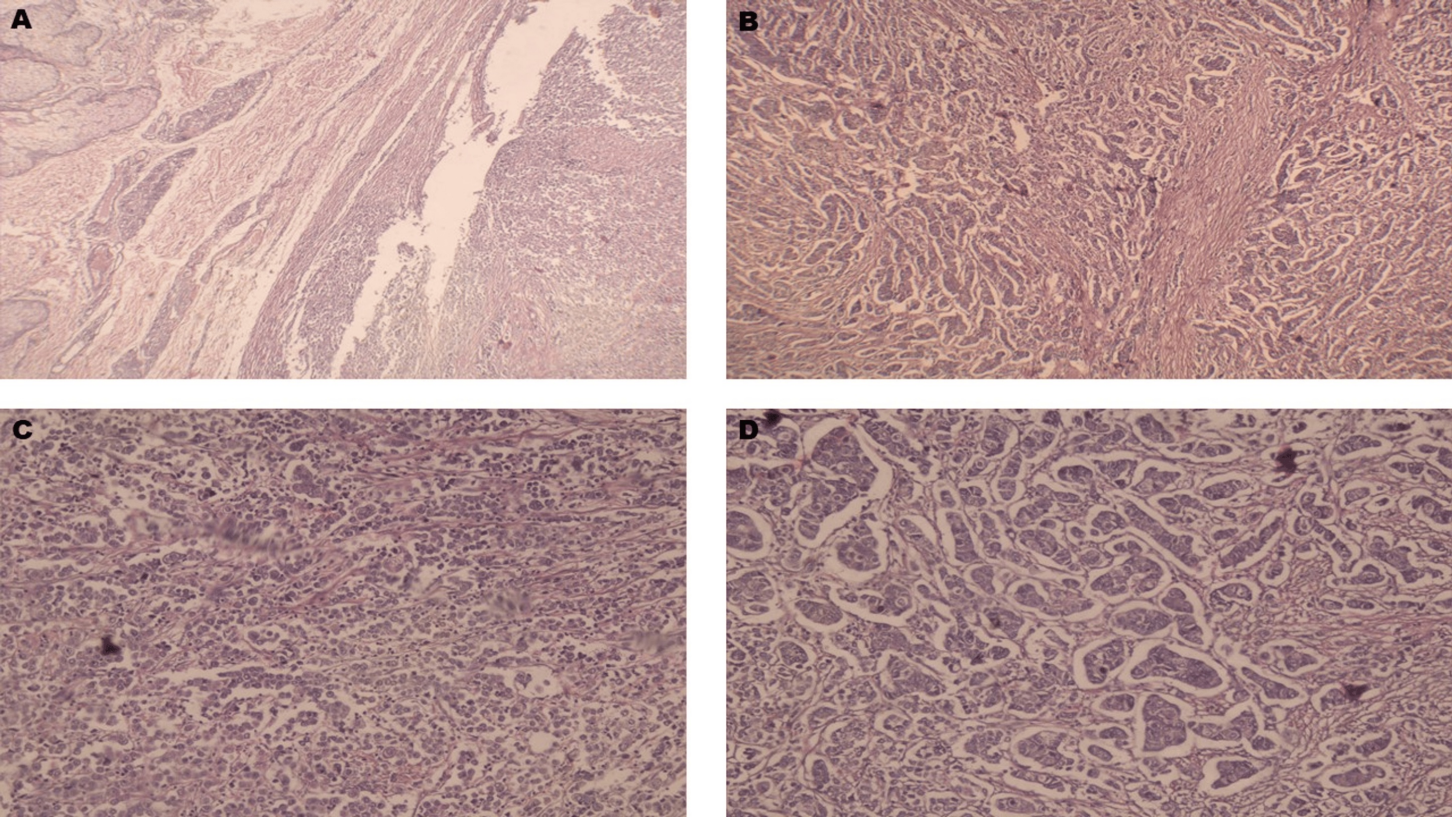
Photomicrographs reveal nodular proliferation of basaloid cells in a cribriform pattern A: H&E stain, 4x magnification of the specimen; B, C, and D: H&E stain, 10x magnification of the specimen H&E: Hematoxylin and eosin

The FNAC reports revealed mucoepidermoid carcinoma of the right submandibular gland. The patient was also advised for USG neck, which revealed multiple enlarged submental, right submandibular, and bilaterally jugulo-digastric lymph nodes. Some of the lymph nodes showed necrosis. The Mantoux test of the patient showed positive results, which confirmed tuberculosis. The final diagnosis of the lesion was ACC with tuberculosis. Surgical management of the lesion was planned after the final diagnosis. All preoperative investigation reports were normal. Wide local excision of the lesion was done under general anesthesia, up to level V lymph nodes, and reconstruction was done with the pectoralis major myocutaneous flap of the right side. The excised specimen was sent for histopathological examination, which revealed nodular proliferation of basaloid cells in a cribriform pattern (Figure 4).

**Figure 5:**
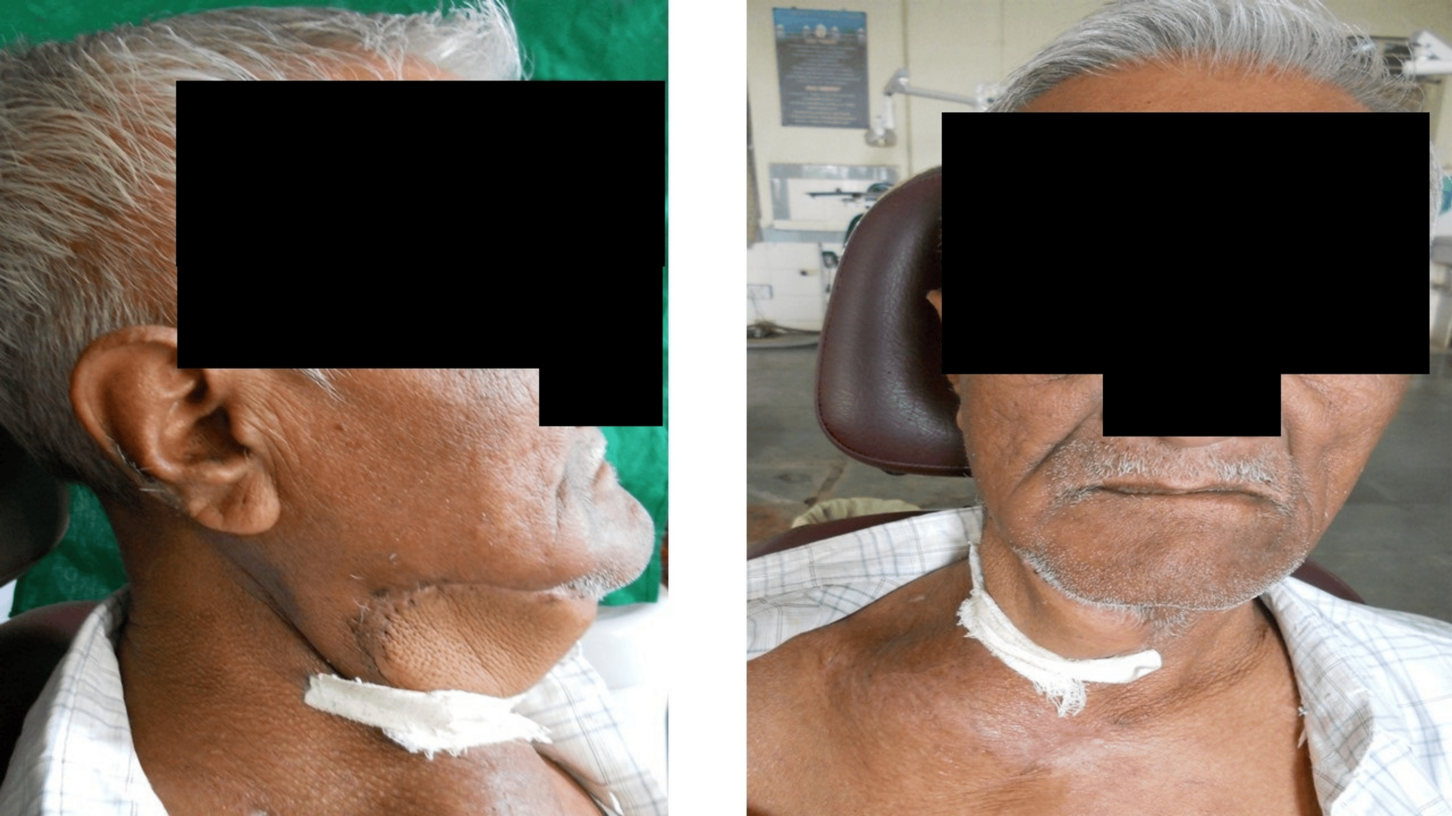
Photographs of the patient at the one-year follow-up show a healthy donor and recipient site

The patient was advised to avoid trauma to the surgical site. After the surgery, the patient was prescribed tablet Chymoral Forte (trypsin and chymotrypsin enzyme) twice a day, capsule amoxicillin 500 mg thrice a day, tablet Voveran (diclofenac) 50 mg twice a day, and tablet Rantac (ranitidine) 150 mg twice a day for five days. The patient was kept on a high-protein diet and was advised to maintain oral hygiene using betadine mouthwash. The initial empiric treatment for tuberculosis following the four-drug regimen was given to the patient: tablet isoniazid 600 mg once a day for three days a week, capsule rifampicin 400 mg once a day for three days a week, tablet ethambutol 1200 mg once a day for three days a week, and tablet pyrazinamide 1500 mg once a day for three days a week. Tablet Emset 4 mg (ondasetron) was advised 15 minutes before the antiretroviral therapy. The regimen was continued for two months. The patient was recalled for follow-up after three months of the surgery. The surgical site was healthy with no dehiscence or gaping and no bleeding. The mouth opening of the patient was adequate. Both the donor site and recipient site were healthy. The patient was asymptomatic at the one-year follow-up, which revealed no evidence of recurrence (Figure 5).

## Discussion

Billroth initially referred to ACC as "cylindroma" in 1859. The prognosis is poor, and the overall mortality rate is high for ACC. About 29.6% of small salivary gland tumors are ACCs [12]. Spiro et al. reported 242 salivary gland ACC cases. Of those, 171 patients had lesions affecting accessory glands; 64 patients (26%) had palate lesions, with the tongue being the second most afflicted location [9]. The cases had a modest female prevalence of 66.6%, which is consistent with descriptions seen in international literature [10]. The submandibular gland is typically impacted when ACC occurs in major salivary glands, while minor salivary glands account for the majority of cases [7]. When ACC is histomorphologically examined, epithelial and myoepithelial cells are frequently seen in a variety of morphologic patterns, such as tubular, cribriform, and solid patterns. Basaloid squamous cell carcinoma, pleomorphic adenoma, basal cell adenocarcinoma, basal cell adenoma, and epithelial-myoepithelial carcinoma are among the basaloid benign and malignant entities that can be identified in the differential diagnosis of ACC [13-15].

Four different treatment techniques are available for ACC: surgical therapy, radiation therapy, chemotherapy, and combination treatments. Since only surgical excision or radiation therapy eliminates the chance of recurrence in the margins or metastases, which often occur in the cervical lymph nodes, lungs, bones, and brain, it has been the treatment of choice in the majority of patients [4]. When paired with complementary therapeutic procedures, oncologic surgical operations for tumor excision result in patients' features being temporarily or permanently disfigured, resulting in physiognomy deformities and functional alterations. If surgery is not an option, prosthetic rehabilitation is recommended to enhance the patient's cosmetic appearance, address eating, swallowing, and speech challenges, facilitate social and professional reintegration, and generally improve quality of life [14]. This paper presents the case of a patient with a final diagnosis of ACC with tuberculosis and highlights the clinical, radiographic, and histopathological features and treatment plan of the lesion.

## Conclusions

The rare tumor of the head and neck known as ACC has an indolent history, but it also has a propensity for perineural invasion, disease recurrence, and frequently distant metastases. The primary treatment is surgical resection, usually accompanied by adjuvant radiation to enhance locoregional management. When a patient is not considered ideal for surgery, the procedure is too morbid, or a gross, complete resection is doubtful, definitive radiation treatment is advised. In the present case, surgical excision of the lesion resolved this indolent salivary gland tumor. The current case is noteworthy due to its emphasis on the dearth of information on salivary gland ACCs as they advance. This report emphasizes that individual benefits of multimodality therapy be taken into account and advocates for routine follow-up imaging.
